# Editorial: Beyond the plate: the role of diet and the microbiota-gut-brain axis in neurodegenerative diseases

**DOI:** 10.3389/fnut.2026.1894989

**Published:** 2026-07-01

**Authors:** Sávio Henrique de Cicco Sandes, Dionisio Pedro Amorim-Neto

**Affiliations:** Department of Food Science and Nutrition, Faculty of Food Engineering, University of Campinas, Campinas, Brazil

**Keywords:** Alzheimer's disease, bioactive compounds, diet and nutrition, dietary interventions, microbiota-gut-brain axis, neurodegenerative diseases, Parkinson's disease, prebiotics and probiotics

The studies included in this Research Topic collectively reinforce the concept that diet and microbiota-related mechanisms are deeply intertwined with neurodegenerative processes. Importantly, these contributions extend beyond descriptive associations and explore mechanistic pathways, translational opportunities, and emerging therapeutic strategies involving nutritional compounds, dietary patterns, microbial modulation, and systemic metabolism.

One important theme across this Research Topic is the influence of dietary patterns on neurodegenerative risk. Xian et al. investigated the association between plant-based dietary patterns and dementia prevalence among Chinese older adults using data from a large longitudinal cohort. Their findings demonstrated that healthier plant-based dietary indices were associated with lower dementia prevalence, whereas unhealthful plant-based dietary patterns showed the opposite relationship. These observations reinforce the growing recognition that overall dietary quality, rather than isolated nutrients alone, may substantially influence cognitive aging and dementia susceptibility.

Complementing these epidemiological findings, Chen et al. explored the neuroprotective effects of coconut oil-derived medium-chain triglycerides (MCTs) in 5 × FAD mice. Their study demonstrated that MCT supplementation ameliorated memory deficits, reduced amyloid pathology, promoted neurite outgrowth, and improved gut homeostasis. Notably, the authors also identified alterations in gut microbiota composition, including increased abundance of *Akkermansia*, supporting the idea that dietary lipids can influence neurodegeneration through microbiota-mediated pathways. Together, these findings highlight the therapeutic potential of dietary interventions capable of simultaneously targeting metabolism, neuronal integrity, and gut microbial ecology.

Another major focus of this Research Topic is the identification of bioactive compounds with neuroprotective properties. Shi et al. investigated bavachalcone in an MPTP-induced mouse model of Parkinson's disease (PD) and demonstrated improvements in motor function, preservation of dopaminergic neurons, reduced neuroinflammation, and modulation of both gut microbiota composition and systemic metabolism. Their work further supports the emerging paradigm that microbiota-associated metabolic pathways are closely linked to neurodegenerative progression and may represent viable therapeutic targets.

Similarly, Zhao et al. reviewed the therapeutic potential of hesperidin, a citrus-derived flavonoid, emphasizing its antioxidant, anti-inflammatory, epigenetic, and neuroprotective properties. Beyond discussing classical mechanisms involving oxidative stress and inflammation, the review highlighted how hesperidin may influence synaptic function, mitochondrial homeostasis, and blood-brain barrier integrity. Importantly, the authors also addressed translational limitations, including poor bioavailability and restricted blood-brain barrier permeability, underscoring the need for improved delivery systems and clinically relevant formulations.

The importance of the microbiota-gut-brain axis as a therapeutic framework is further reinforced in the review by Auclair-Ouellet et al., which comprehensively examined microbiome-based interventions across several neurodegenerative disorders, including Alzheimer's disease, PD, Amyotrophic Lateral Sclerosis, Multiple Sclerosis, and Huntington's disease. The authors summarized evidence supporting the use of probiotics, prebiotics, synbiotics, and postbiotics to modulate neuroinflammation, intestinal permeability, microbial metabolites, and disease-associated protein aggregation. Importantly, the review also identified critical knowledge gaps, particularly regarding the translation of promising preclinical findings into robust clinical outcomes.

Finally, Sun et al. provided a bibliometric perspective on gastrointestinal dysfunction in PD, revealing the rapid expansion of research focused on the brain-gut-microbiome axis over the last decade. Their analysis identified major research frontiers centered on α-synuclein pathology, gastrointestinal symptoms, and microbiota-targeted interventions such as probiotics and fecal microbiota transplantation. This bibliometric mapping not only highlights the increasing scientific interest in gut-related mechanisms in PD but also demonstrates how the field has progressively shifted toward integrative and systems-level approaches.

Collectively, the contributions assembled in this Research Topic illustrate the growing convergence between nutrition science, microbiology, immunology, metabolism, and neuroscience. They reinforce the concept that neurodegenerative diseases should no longer be viewed solely as disorders of the brain, but rather as systemic conditions involving complex interactions between diet, microbial communities, peripheral physiology, and neural function.

Although substantial progress has been made, several challenges remain. Many microbiota-related findings are influenced by interindividual variability, dietary habits, medication use, geography, and methodological differences across studies. Furthermore, mechanistic insights derived from experimental models still require validation in large and well-controlled human studies. Future research will therefore benefit from standardized methodologies, longitudinal clinical investigations, and multi-omics approaches capable of integrating microbial, metabolic, immunological, and neurological data.

Nevertheless, the studies presented in this Research Topic provide compelling evidence that nutritional strategies and microbiome-targeted interventions may represent promising complementary approaches for preventing or mitigating neurodegenerative diseases. By moving “beyond the plate,” this field continues to redefine how we understand the relationship between diet, the gut microbiota, and brain health, opening new possibilities for precision nutrition and personalized neuroprotective therapies.

